# Endovascular thrombectomy is cost-effective in acute basilar artery occlusion stroke

**DOI:** 10.3389/fneur.2023.1185304

**Published:** 2023-04-26

**Authors:** Julian Schwarting, Sebastian Rühling, Jannis Bodden, Stéphanie K. Schwarting, Claus Zimmer, Dirk Mehrens, Jan S. Kirschke, Wolfgang G. Kunz, Tobias Boeckh-Behrens, Matthias F. Froelich

**Affiliations:** ^1^Department of Diagnostic and Interventional Neuroradiology, Klinikum Rechts der Isar, Technical University Munich, Munich, Germany; ^2^Institute for Stroke and Dementia Research (ISD), University Hospital, LMU Munich, Munich, Germany; ^3^Department of Medicine I, University Hospital, LMU Munich, Munich, Germany; ^4^Department of Radiology, University Hospital, LMU Munich, Munich, Germany; ^5^Department of Radiology and Nuclear Medicine, University Medical Center Mannheim, University of Heidelberg, Mannheim, Germany

**Keywords:** stroke, cost-effectiveness, basilar artery occlusion, thrombectomy, endovascular treatment

## Abstract

**Objective:**

Endovascular thrombectomy is a long-established therapy for acute basilar artery occlusion (aBAO). Unlike for anterior circulation stroke, cost-effectiveness of endovascular treatment has not been evaluated and is urgently needed to calculate expected health benefits and financial rewards. The aim of this study was therefore to simulate patient-level costs, analyze the economic potential of endovascular thrombectomy in patients with acute basilar artery occlusion (aBAO), and identify major determinants of cost-effectiveness.

**Methods:**

A Markov model was developed to compare outcome and cost parameters between patients treated by endovascular thrombectomy and patients treated by best medical care, based on four recent prospective clinical trials (ATTENTION, BAOCHE, BASICS, and BEST). Treatment outcomes were derived from the most recent literature. Uncertainty was addressed by deterministic and probabilistic sensitivity analyses. Willingness to pay per QALY thresholds were set at 1x gross domestic product *per capita*, as recommended by the World Health Organization.

**Results:**

Endovascular treatment of acute aBAO stroke yielded an incremental gain of 1.71 quality-adjusted life-years per procedure with an incremental cost-effectiveness ratio of $7,596 per QALY. This was substantially lower than the Willingness to pay of $63,593 per QALY. Lifetime costs were most sensitive to costs of the endovascular procedure.

**Conclusion:**

Endovascular treatment is cost-effective in patients with aBAO stroke.

## Introduction

Stroke remains the leading cause of long-term disability worldwide and the second most common cause of death despite significant advances in therapy (1). A severe subtype of stroke is acute basilar artery occlusion (aBAO) which accounts for approximately 10% of ischemic strokes caused by intracranial large-vessel occlusion (2).

Affected patients suffer in up to 80% from severe disability or die, despite best medical care (3). Although many patients have been treated by endovascular thrombectomy (EVT) even before it became a standard therapy for the anterior circulation, there have been no prospective randomized trials showing the benefit of endovascular thrombectomy in patients with aBAO until recently (2–4).

Four multicenter, prospective, randomized, controlled trials of endovascular thrombectomy for aBAO were published in 2019–2022: ATTENTION (3), BAOCHE (5), BASICS (6), and BEST (7). Despite regional biases and a heterogeneity in outcome, time windows and thrombolysis rates, these trials provide high-level evidence for improvement of functional outcomes and independence in patients treated with EVT (8).

Cost-effectiveness of EVT after large-vessel occlusions in the anterior circulation was extensively investigated and results in long-term cost-savings for healthcare systems and societies, for instance in the United States, where estimated cost savings are approximately $40 billion/year and are predicted to increase substantially within the next decade (9–12).

To our knowledge, there is no evidence of cost-effectiveness of endovascular thrombectomy for acute basilar artery occlusion. As these data are urgently needed to calculate expected health benefits and financial rewards, for instance for further developments of endovascular treatments, we defined and quantified public health and cost consequences of endovascular treatments for aBAO stroke patients and healthcare systems based on the recent literature.

## Materials and methods

### Study selection

To simulate long-term costs and outcomes of patients, we selected all published multicenter, prospective, randomized controlled trials published by the end of 2022, as also identified by a meta-analysis of Malik et al. (8): ATTENTION (3), BAOCHE (5), BASICS (6), and BEST (7) (Table 1).

**Table 1:** Included clinical trials.

		ATTENTION (3)	BAOCHE (5)	BASICS (6)	BEST (7)
Date		2021–2022	2016–2022	2011–2019	2015–2017
Symptom onset to inclusion		0–12	06–24	0–6	0–8
Screened		340	217	424	288
Participants	Total:	340	202	300	131
	Intervention:	225	102	154	77
	Best medical care:	115	100	146	54
*n* (%) mRS0	Intervention:	11 (4.9)	7 (6.9)	8 (5.2)	7 (9.1)
	Best medical care:	5 (4.3)	1 (1.0)	6 (4.1)	3 (5.6)
*n* (%) mRS1	Intervention:	34 (15.1)	19 (18.6)	19 (12.3)	16 (20.8)
	Best medical care:	5 (4.3)	6 (6.0)	13 (8.9)	5 (9.3)
*n* (%) mRS2	Intervention:	29 (12.9)	16 (15.7)	27 (17.5)	7 (9.1)
	Best medical care:	3 (2.6)	8 (8.0)	25 (17.1)	2 (3.7)
*n* (%) mRS3	Intervention:	29 (12.9)	8 (7.8)	14 (9.1)	6 (7.8)
	Best medical care:	14 (12.2)	11 (11.0)	11 (7.5)	3 (5.6)
*n* (%) mRS4	Intervention:	11 (4.9)	9 (8.8)	10 (6.5)	6 (7.8)
	Best medical care:	6 (5.2)	19 (19.0)	16 (11.0)	11 (20.4)
*n* (%) mRS5	Intervention:	27 (12.0)	12 (11.8)	17 (11.0)	12 (15.6)
	Best medical care:	19 (16.5)	14 (14.0)	12 (8.2)	6 (11.1)
*n* (%) mRS6	Intervention:	84 (37.3)	31 (30.4)	59 (38.3)	23 (29.9)
	Best medical care:	63 (54.8)	41 (41.0)	63 (43.2)	24 (44.4)

For economic modulation, we used only published data. Ethical approval or patient consent was therefore not obtained.

### Economic model structure

**Figure 1:**
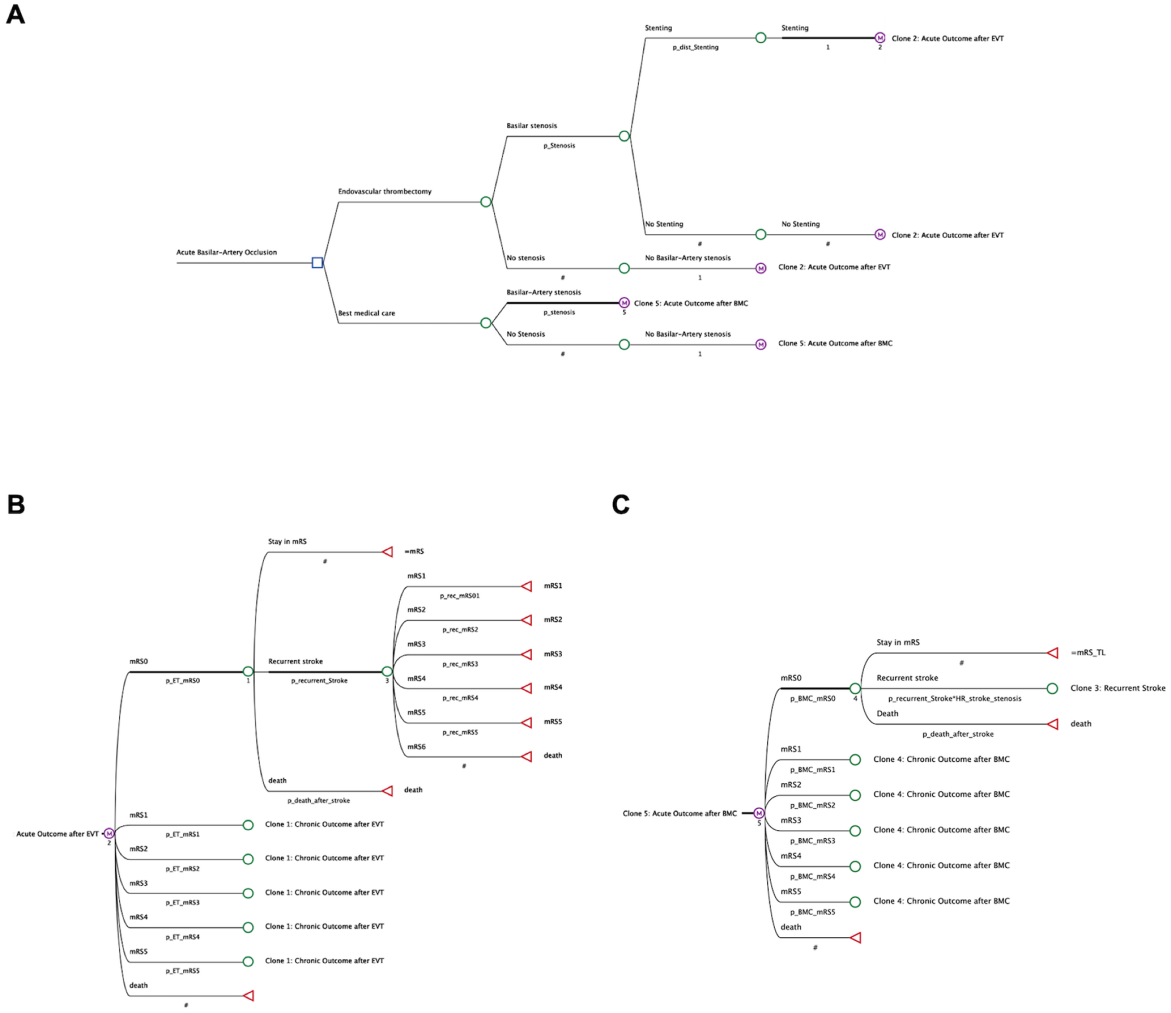
Structure of decision tree and markov-model. **(A)** Patients with acute basilar artery occlusion received either best medical care (BMC) or endovascular thrombectomy (EVT). Patients were then differentiated according to underlying basilar stenosis and stent implantation. The adjacent Markov model simulates lifelong pathways of stroke patients with a possible level of disability, according to the clinical outcomes reported after EVT **(B)** or BMC **(C)**. Costs and effectiveness (QALYs) are compared for both treatment strategies.

To compare endovascular therapy to conventional care from a healthcare perspective, we investigated quality-adjusted life years (QALYs) and costs related to healthcare providers in the United States with a Markov-decision-model, designed in accordance with the Consolidated Health Economic Evaluation Reporting Standards (CHEERS; Figure 1) (13). Economic modeling was conducted with the decision-analytic software TreeAge Pro 2022 (TreeAge, Williamstown, MA, United States) based on a cycle length of 1 year.

Patients entered the model after diagnosis of acute basilar artery occlusion at a stroke center before the decision between endovascular thrombectomy (EVT) and best medical care (BMC) at an assumed age of 66 years. This is the average patient age in the included prospective trials (3, 5–7).

.In the decision model, we differentiated patients with acute-onset basilar occlusion in four total cohorts, depending on the method of treatment (EVT or BMC only) and on occurrence of an underlying intracranial atherosclerosis with an increased probability of recurrent stroke (14). Patients who received endovascular therapy were then further categorized depending on their need of stent implantation. (Figure 1A).

In our Markov model, we simulated patients’ long-term outcome that could either stay on the level of disability reported after 90 days, or could be deteriorated by occurrence of further strokes, resulting in the same or a lower mRS state, or death. (Figures 1B,C) After endovascular thrombectomy with stent placement, we also considered an increased risk of in-stent thrombosis. (Figure 1B).

### Model input parameters

Input parameters were derived from peer-reviewed literature in accordance with international recommendations on the methodological framework of cost-effectiveness analyses and are shown in Table 2 (13, 24).

**Table 2:** Model input data.

Model input	Base-case value	Distribution	Source
Assumed WTP per QALY	$63.593	-	McDougal (15)
Starting age	66 years	-	Assumption
Discount rate	3.00%	-	Assumption
Costs			
Stroke hospitalization costs (mRS 0–2/3–5/6)	$26,705/$106,533/$88,159	γ	Mu (16)
Costs of first 90 days after discharge (mRS 0–2/3–5)	$15,213/$26,496	γ	
Total costs day 91–365 after stroke (mRS 0–2 and 3–5)	$24,988/$35,140	γ	
Additional cost EVT	$17,103	γ	Shireman (12)
Additional cost stent implantation	$9,000	γ	Stryker, United States
Acute care costs recurrent stroke	$29,259	γ	Chambers (17)
Long-term annual cost after stroke for mRS 0/1/2/3/4/5	$14,230/$14,653/$16,952/$29,107/$58,913/$86,612	γ	Shireman (12)
Initial probabilities			
Underlying basilar stenosis in acute basilar artery occlusion	0.397 ± 0.107	-	Summarized results of ATTENTION, BAOCHE, BASICS, BEST (3, 5–7)
Functional outcome 90d after EVT mRS 0/1/2/3/4/5/6	6.5/16.5/14.0/9.5/7.0/12.75/33.75	-	
Functional outcome 90 days after BMC mRS 0/1/2/3/4/5/6	3.75/7.0/8.0/9.25/13.75/12.5/45.75	-	
Acute stenting of observed basilar stenosis in initial EVT	0.618	-	
Transition probabilities			
Stroke recurrence (year 1–10)	0.059/0.036/0.025/0.022 / 0.022 / 0.027 / 0.027 / 0.023 / 0.028 / 0.016	-	Pennlert (18)
Annual death hazard ratios for survivors mRS 0/1/2/3/4/5	0.129/0.136/0.164/0.247/0.135/0.189	-	Hong (19)
Outcome after recurrent stroke in mRS 0 or 1/2/3/4/5/6	0.129/0.136/0.164/0.247/0.135/0.189	-	Goyal (20)
Age-adjusted mortality	U.S. life tables	-	Arias (21)
RR for stroke recurrence with underlying basilar stenosis	3.4	-	Gulli (14)
HR for in-stent thrombosis	1.057	-	Riedel (22)
Utilities			
Quality of life (mRS Score 0–5)	1.00/0.91/0.76/0.65/0.33/0.00/0.00	β	Chaisinanukul (23)

#### Initial and transition probabilities

Initial outcome probabilities for functional outcome 90 days after acute basilar occlusion, for basilar stenosis and stent implantation, were derived from the recently published data of the four selected trials ATTENTION (3), BAOCHE (5), BASICS (6), and BEST (7) (Table 1).

Transition Probabilities used for long-term modulation of time-dependent stroke recurrence rates (12) and outcomes were estimated based on control cohort from the HERMES-dataset (18). For the cohorts with underlying basilar stenosis and/or implanted stents, we adapted the risks accordingly (25). The probability of death after stroke at a specific functional outcome state was calculated by multiplication of hazard ratios of death at the specific functional outcome (10) with age-specific average background mortality rates, according to U.S. life tables (21).

#### Costs

The perspective of the U.S. healthcare system was adopted to calculate cumulative discounted costs in U.S.$. All costs were adjusted to 2022 values using a discount rate of 3%/year.

Hospital costs for acute stroke care as well as post-hospitalization costs within the first 365 days were included from a nation-wide cost analysis of acute stroke care costs by Mu et al. (16) Costs for EVT as well as long-term healthcare costs of stroke survivors were estimated according to a previous long-term projection of a patient cohort of *n* = 428 (12). Acute care costs of recurrent strokes were estimated based on Chambers et al. (17) Costs of additional stent placement was estimated, based on costs of the Neuroform Atlas^®^ Stent (Stryker, United States). For sensitivity analysis, all costs were modulated using γ-distributions.

#### Utilities

Outcomes were simulated in terms of quality-adjusted life years (QALYs), calculated as life spent in specific mRS-States multiplied by quality of life (range: 0–1) which were acquired from a meta-analysis of 11 stroke intervention trials (23). For sensitivity analysis, all utilities were modulated using β-distributions.

### Cost-effectiveness analysis

Treatment strategies were compared in incremental costs, incremental effectiveness, and incremental cost-effectiveness ratios (ICERs).

According to WHO-CHOICE recommendations, we set the willingness to pay (WTP) to 1x the country-specific gross domestic product (GDP) *per capita* ($ 63.593, highly cost-effective) and 3x the country-specific GDP *per capita* ($ 190.779, cost-effective) (15). Resulting thresholds for the United States were based on 2020 data from the World Bank (26). All costs and outcomes were discounted by 3% annually, as recommended by consensus (24).

### Sensitivity analysis

Deterministic and probabilistic sensitivity analyses were performed to analyze the impact of uncertainty:

Deterministic cost sensitivity analysis was conducted to reveal the influence of individual cost variables of the model depending on single input parameters. Variations of +/− 25% of base costs were used as range.
Probabilistic sensitivity analysis was used to adjust several input parameters based on their probability distributions and simulate the model results in 30,000 Monte Carlo simulations.

## Results

### Cost-effectiveness analysis

In the base-case scenario, best medical care resulted in average discounted outcomes of 2.58 QALYs, 95% CI [2.58, 2.58] QALYs per patient over a life-time horizon, whereas endovascular thrombectomy resulted in 4.29 QALYs, 95% CI [4.28, 4.29] QALYs. Average discounted lifetime-costs summed up to $313,550, 95% CI [$313,405, $313,695] after BMC and $326,570, 95% CI [$326,432, $326,708] after EVT. This resulted in an incremental cost-effectiveness ratio of $7,595 per QALY. Therefore, endovascular thrombectomy showed a higher effectiveness compared to best medical care. Additional expenses per QALY were $55,998 cheaper than the highly cost-efficient willingness to pay for an extra QALY. Interestingly, however, we found that patients with an underlying basilar stenosis generated lower lifetime costs when treated by EVT. Here, EVT was the dominant strategy with lower costs and better outcome for this patient cohort. In a separate analysis of patients presenting delayed after onset, which were specifically investigated in the BAOCHE trial, we detected cost-saving effects with improvements from 2.56 to 4.68 QALYS and a cost reduction by $13,172. (Table 3).

**Table 3:** Cost-effectiveness analysis.

	Cohort	Costs	Incr. Costs	Effectiveness (QALY)	Incr. Effectiveness (QALY)	ICER ($/QALY)
Best medical care	*Total*	$313,550 [$313,405, $313,695]	–	2.58 [2.58, 2.58]	–	–
	*Basilar stenosis*	$337,716	–	2.22	–	–
	*No basilar stenosis*	$297,641	–	2.81	–	–
Endovascular thrombectomy	*Total*	$326,570 [$326,432, $326,708]	$12,979	4.28 [4.28, 4.29]	1.71	$7,596
	*Basilar stenosis; Stenting*	$334,570	-$3,146	4.26	2.04	Dominance
	*Basilar stenosis; No stenting required*	$324,005	-$13,711	4.29	2.22	Dominance
	*No basilar stenosis*	$324,005	$26,364	4.29	1.48	$17,813
Onset: 6 – 24 h (BAOCHE)	*BMC*	$347,815 [$344,153, $351,478]	–	2.56 [2.51, 2.60]		
	*EVT*	$334,643 [$331,594, $337,691]	-$13,172	4.68 [4.62, 4.74]	2.12	Dominance

### Probabilistic sensitivity analysis

**Figure 2:**
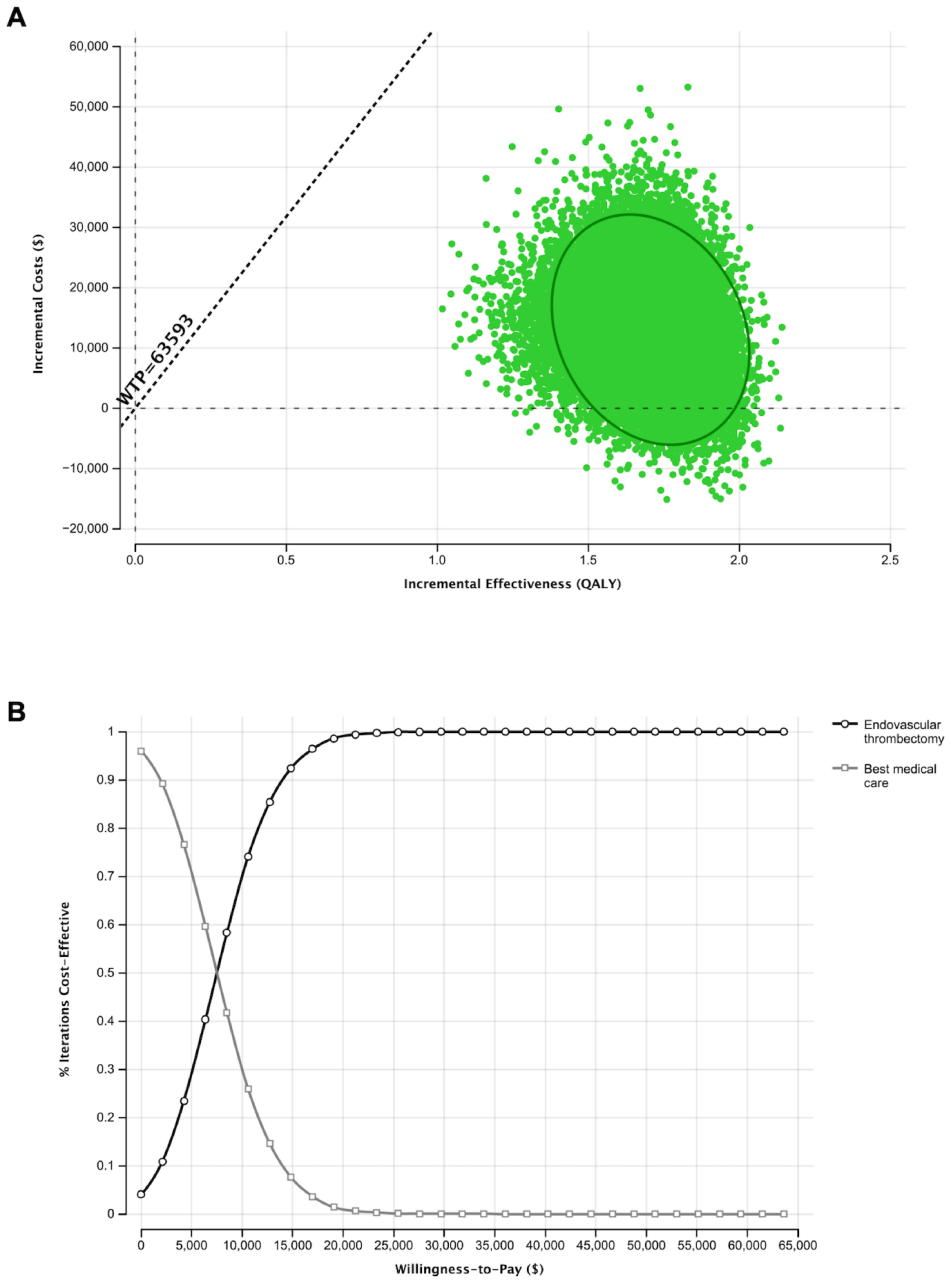
Probabilistic sensitivity analysis and Monte Carlo Simulation with 30,000 iterations for the base-case scenario. **(A)** Results of 30,000 simulations of incremental costs and incremental outcomes of endovascular thrombectomy in comparison to best medical care. **(B)** The Acceptability curve shows the cost-effectiveness of both therapeutic depending on an increasing willingness to pay.

In probabilistic sensitivity analysis, at a WTP threshold of $ 63,593 per QALY, EVT was cost-effective in 100% of iterations. Interestingly, no iterations showed a decrease effectiveness, while a substatial number of iterations were observed with an increased effectiveness and lower costs (Figure 2A). Above a WTP threshold of $5,795/Qualy, EVT is the cost-effective alternative in the majority of iterations (Figure 2B).

### Deterministic sensitivity analysis

**Figure 3:**
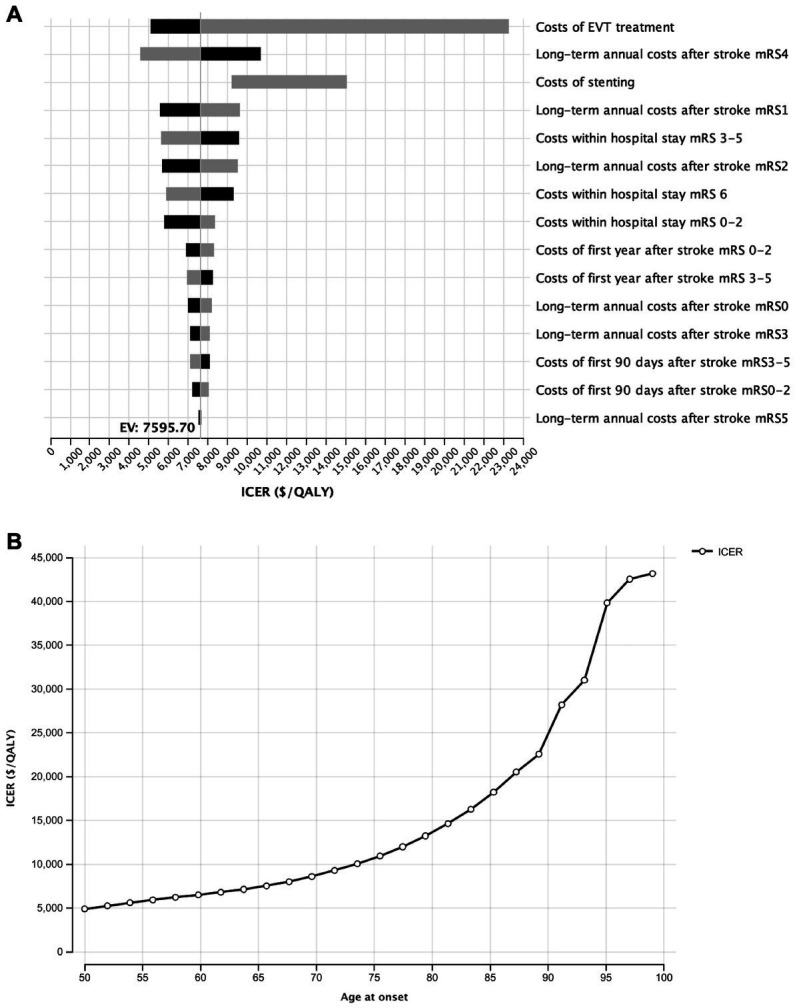
Influence of costs variations for the model outcome. **(A)** Deterministic sensitivity analysis of the impact of all cost variations on the model outcomes upon variation by 25%. **(B)** One-Way sensitivity analysis of the influence of age at onset on the incremental cost-effectiveness.

To account for the differing costs of stroke outcomes and procedures in different healthcare systems, a deterministic sensitivity analysis of all implemented costs with a range of +/− 25% was performed. Here, cost-effectiveness was most sensitive to costs of endovascular treatment. All further cost variations did not result in an increase of the ICER to > $25,000/QALY. All costs remained below the WTP threshold, indicating the cost-effectiveness endovascular thrombectomy in the shown setting. (Figure 3A).

Additionally, we investigated the influence of age at the aBAO on the ICER in the wide age range from 55 to 80 years. EVT was most cost-efficient at an age of 50 years (ICER: $4,909/QALY). Although ICER increased to $43,182/QALY at the age of 99 years, cost-effectiveness was given independently from age (Figure 3B).

## Discussion

Our study represents the first cost-effectiveness-analysis of endovascular thrombectomy after acute basilar occlusion (aBAO) and optional basilar artery stenting based on only recently published prospective, randomized, multicenter trials (3, 5–7). In our simulation, lifetime costs of an aBAO were $313,631 with best medical care and rose to $326,610 with additional endovascular thrombectomy. With an ICER of $7,596 per additional QALY, EVT was thereby cost-efficient when compared with a willingness to pay of $63,593. In patients who present 6–24 h after onset and patients with underlying ICAD, EVT was cost-saving, i.e., dominant.

In anterior circulation stroke, cost-effectiveness of endovascular thrombectomy has been demonstrated in several studies, most recently also in patients with underlying with preexisting disabilities and low ASPECT scores (11, 27, 28). In data from the HERMES collaboration (Highly Effective Reperfusion Evaluated in Multiple Endovascular Stroke Trials), thrombectomy was previously reported with lifetime cost savings of $23,203 and an outcome improvement of 6.79 vs. 5.05 QALYs in comparison to BMC (12).

The capability of endovascular thrombectomy to prevent severe neurological deficits is, however, lower than in anterior-circulation stroke and was quantified to 1.69, 95% CI [1.05–2.71] in BAO (8), vs. 2.47, 95 CI [1.79, 3.41] in the anterior circulation (20).

Deterministic sensitivity analysis, showed that predominantly costs of the intervention itself, estimated at $17,103, had a significant impact on the ICER, but did not rise the ICER above the WTP threshold. Overall lifetime costs were $12,979 higher with endovascular therapy, which implies that additional costs of thrombectomy are partially compensated by the excessive lifetime costs of reduced functional outcome. Further cost drivers are long-term annual costs after stroke, predominantly in patient with severe neurological deficits (mRS4 and 5). High additional interventional costs of intracranial stents, frequently used in the posterior circulation due to high rates of intracranial atherosclerosis, do not influence cost-effectiveness (14).

## Limitations

1. Our simulation represents simplified linear diagnostic and therapeutic pathways, which are limited by the quality and validity of its input variables. Due to limited data availability on patients with aBAO, several variables, such as costs of interventions, quality of life data, and long-term costs had to be taken from studies primarily investigating anterior circulation stroke. Group imbalances and partly retrospective evaluations may additionally limit validity of model inputs.
2. Although inclusion criteria and results varied between studies, we selected all recently published trials ATTENTION (3), BAOCHE (5), BASICS (6), and BEST (7) for input data to have the broadest spectrum of evidence for endovascular thrombectomy after aBAO.

Each of the input studies has, however, limitations which may affect its results and thereby also cost-effectiveness:

Basilar artery international cooperation study (BASICS) showed no significant benefit of EVT in comparison to BMC. This could have probably been caused by an overrepresented inclusion of clinically minor strokes as patient recruitment was prolonged and inclusion criteria had to be adapted eventually (6, 8).
BEST (basilar artery occlusion endovascular intervention vs. standard medical treatment) investigated patients with high admission NIHSS scores (32 in intervention and 26 in control). However, there was a crossover rate of 13%, mainly in patients randomized from medical therapy to endovascular therapy (7, 8).
ATTENTION (endovascular treatment of acute basilar artery occlusion) study and BAOCHE (basilar artery occlusion Chinese endovascular trial) showed benefits of EVT in time windows of up to 12 h (ATTENTION) or 6–24 h (BAOCHE) after symptom onset. While recruitment time and cross-over was low, intravenous thrombolysis (IVT) was only given in approximately 1/3 of patients, most likely due to delayed onset in patients. Because observational studies demonstrated that shown IVT beyond 4.5 h may benefit BAO patients, differences between groups might be smaller if IVT had been given more frequently (3, 5, 8, 29).
Three out of four of these studies have been conducted in China. This is of particular relevance as the Asian population is known to have a higher prevalence of intracranial atherosclerosis in comparison with a Western population (8, 30).

1. Results were calculated based on U.S. data and are therefore not generalizable to other countries. However, the simulation can compensate cost differences of up to 25%, as shown in Figure 3.

## Conclusion

Although associated with an increase of lifetime costs by $12,979 per patient, endovascular thrombectomy of acute basilar occlusion is cost-effective in the United States with an ICER of $7,595 per QALY. In patients with underlying ICAD and patients with presentation 6–24 h after onset, endovascular thrombectomy is a dominant treatment strategy with lower lifetime costs and better outcome. Generated lifetime costs are most sensitive for costs of the intervention.

## Data availability statement

The original contributions presented in the study are included in the article/supplementary material, further inquiries can be directed to the corresponding author.

## Author contributions

JS, TB-B, and MF contributed to conception and design of the study. JS, SR, JB, DM, and SS contributed with data collection. JK and WK helped in interpreting the results. JS wrote the first draft of the manuscript. All authors contributed to the article and approved the submitted version.

## Conflict of interest

The authors declare that the research was conducted in the absence of any commercial or financial relationships that could be construed as a potential conflict of interest.

## Publisher’s note

All claims expressed in this article are solely those of the authors and do not necessarily represent those of their affiliated organizations, or those of the publisher, the editors and the reviewers. Any product that may be evaluated in this article, or claim that may be made by its manufacturer, is not guaranteed or endorsed by the publisher.
